# Mediterranean diet and osteoarthritis: an update

**DOI:** 10.1007/s40520-024-02883-8

**Published:** 2024-12-03

**Authors:** Nicola Veronese, Francesco Saverio Ragusa, Ligia J. Dominguez, Claudia Cusumano, Mario Barbagallo

**Affiliations:** 1 Department of Health Promotion, Mother and Child Care, Internal Medicine and Medical Specialties, University of Palermo, Palermo, Italy; 2 Department of Medicine and Surgery, Kore University of Enna, Piazza dell’Università, Enna, 94100 Italy

**Keywords:** Mediterranean diet, Osteoarthritis, Inflammation, Oxidative stress, Pain, Older adults

## Abstract

The Mediterranean diet has gained significant attention for its potential health benefits on diverse pathological conditions including osteoarthritis (OA), a prevalent degenerative joint disease characterized by cartilage breakdown and inflammation. Numerous observational studies have suggested that adherence to the Mediterranean diet, may have protective effects against OA. The abundance of antioxidants and anti-inflammatory compounds and omega-3 fatty acids, among the Mediterranean diet components is believed to contribute to its beneficial effects on OA. Research investigating the association between the Mediterranean diet and OA has shown promising results. Several observational studies have reported that adherence to the Mediterranean diet is associated with a reduced risk of developing OA and with lower severity of OA symptoms. Additionally, intervention studies have demonstrated improvements in pain, function, and quality of life among OA patients following a Mediterranean diet intervention. Furthermore, emerging evidence suggests potential mechanisms underlying the protective effects of the Mediterranean diet against OA, including its ability to reduce inflammation, oxidative stress, and cartilage degradation. However, further well-designed randomized controlled trials and mechanistic studies are needed to elucidate the precise mechanisms and establish causality. In conclusion, the Mediterranean diet appears to be a promising dietary approach for the prevention and management of OA. Its rich array of nutrients and bioactive compounds may exert protective effects against OA development and progression, although more research is warranted to confirm these findings and elucidate underlying mechanisms.

**Graphical abstract:**

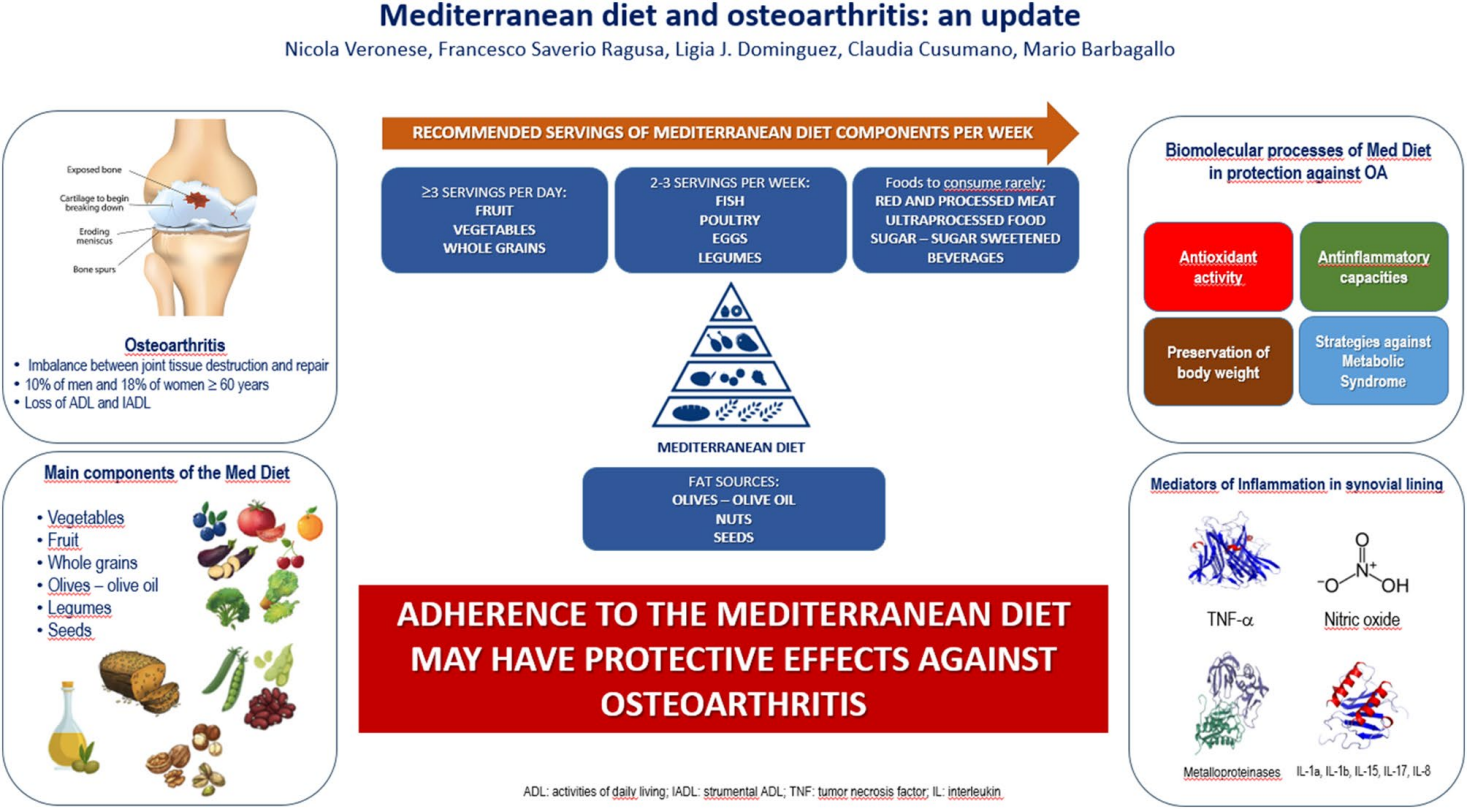

## Introduction

Osteoarthritis (OA) is the most common form of arthritis and a leading cause of disability worldwide, particularly among older adults [1]. With the aging of the global population and rising prevalence of obesity and sedentary lifestyles, the burden of OA is expected to increase substantially in the coming decades [2]. Understanding the epidemiology of OA is essential for informing public health strategies, resource allocation, and preventive interventions aimed at mitigating its impact on individuals and healthcare systems.

Epidemiological studies have provided valuable insights into the prevalence, incidence, risk factors, and burden of OA across different populations and settings. While OA was traditionally considered a disease of aging, it is now recognized that OA affects individuals of all ages, with a significant proportion of cases occurring in middle-aged and younger adults [3]. Furthermore, OA is not limited to the joints but can affect various joints, including the knees, hips, hands, spine, and feet, leading to pain, stiffness, functional impairment, and reduced quality of life [3].

OA is characterized by the deterioration of the joints involving the articular cartilage and lots of the adjacent tissues [4]. It represents a failure of the balance between destruction and reparation of joint tissue, causing the decrease of articular cartilage, renovation of subchondral bone, osteophyte development, ligament laxity and periarticular muscle failing [5].

Since obesity and unhealthy diet are associated with a higher risk of OA, particularly at the lower limbs, in this review we will discuss the importance of Mediterranean diet (MedDiet) in OA, discussing recent literature about this topic. Embracing a healthy eating pattern is essential for both preventing and managing obesity. Of the many dietary strategies available, the Mediterranean Diet (MedDiet) is among the most thoroughly researched. It focuses on whole, nutrient-dense foods and has consistently demonstrated positive effects on weight control, making it a key approach in the fight against obesity. OA is a major contributor to functional impairment and loss of independence in older persons causing difficulties in maintaining their activities of daily living [6]. Due to the widespread occurrence of this disease and its profound effects on patients’ autonomy and standard of living, many studies have delved into the factors linked to the prevalence of OA. While sociodemographic aspects like age and gender have received the most attention, investigations have also scrutinized the correlation between OA and concurrent health conditions, psychological factors [7] and physical activity [8].

Age-related illnesses or their exacerbation are often linked to unhealthy habits and lifestyles [9]. Making changes to daily habits and diet could potentially prevent the onset of these diseases and positively impact their progression, thereby fostering the concept of Healthy Aging, a term coined to denote aging without the burden of disease [10]. The Mediterranean Diet (MedDiet) has proven to be an effective approach in supporting healthy aging. Promoting this lifestyle through health policies appears to be the most effective strategy in reaching this objective [9]. The MedDiet has a low Dietary Inflammatory Index illustrating its anti-inflammatory potential. This dietary pattern beneficially modulates the gut microbiota and immune system, including emerging evidence for efficacy against severe acute respiratory syndrome coronavirus 2 [11].

Since obesity and unhealthy diet are associated with a higher risk of OA, particularly at the lower limbs, in this review we will discuss the importance of MedDiet in OA, discussing recent literature about this topic. OA represent a daily challenge for physicians and it currently lacks a remedy. In clinical settings, it is managed through pharmacological means (such as analgesics, topical and oral non-steroidal anti-inflammatory drugs, NSAIDs) as well as nonpharmacological approaches (including therapeutic exercise, aerobic exercise, assistive devices, and lifestyle adjustments) [11], so adding new updates between correlation between MedDiet and OA could represent relevant fact in term of cure and prevention of this disease.

## Methods

In this narrative non-systematic review, we searched for original articles about MedDiet and OA from database inception to 01st April 2024. We searched in Pubmed, Scopus and Embase using a combination of free terms and MeSH terms in these databases. We excluded in vitro and in animal research, whilst all the other studies investigating MedDiet in OA were considered and discussed.

## Components of the mediterranean diet

The concept of the MedDiet, as we have in mind it today, was initially introduced by the Seven Countries Study, conducted by Keys et al. in the 1950s [12]. This ecological investigation conducted predominantly in deprived, rural populations from Crete (Greece) and southern Italy, provided the foundations for the hypothesis that diet may have health protective effects, e.g. against cardiovascular diseases [13].

It’s noteworthy that the concept of the Mediterranean Diet, introduced by Keys et al. just in the previous century, mirrors the dietary patterns and way of life that Mediterranean basin populations have followed for millennia. Mediterranean region, in fact, has peculiar culinary traditions rich in local products, flavors, aromas, colors, and memories, highlighting taste, contact and respect for nature, and giving special value to preparing, sharing and consuming food together with family and friends [14].

**Fig. 1:**
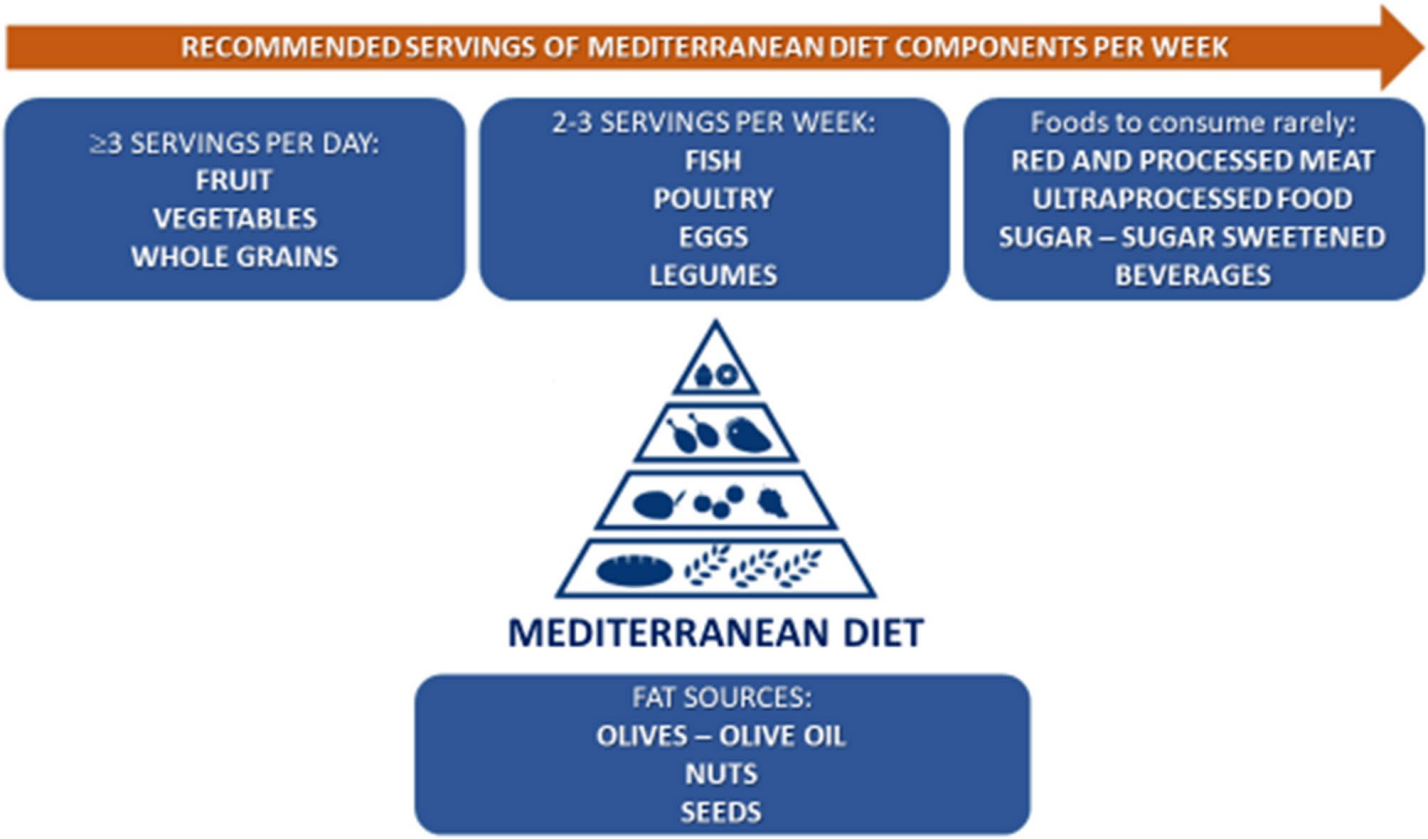
Recommended servings of Mediterranean diet in a week

Figure 1 shows recommended servings for MedDiet in a week, including the consumption of assorted fresh vegetables and fruits three times a day; two to four tablespoon of olive oil consumed daily as main (and almost only) source of dietary fat for seasoning and cooking; regular consumption of nuts and seeds three times a week; legumes consumed several times per week; consumption of whole grains daily; fish and seafood consumed 2 to 3 times per week; consumption of dairy especially yogurt; small portions of cheese consumed less frequently; consumption of 2 to 4 eggs per week; and use of herbs and spices in the preparation of meals to season them. Sweets and red/processed meat are less frequently consumed in this eating model (once a week). The main beverage in the traditional MedDiet is water; wine is consumed in small portions always with meals.

From a biomolecular point of view, MedDiet has several anti-inflammatory effects [15] and could modulate mitochondria processes [16]. Therefore, the composition of MedDiet makes it ideal for the prevention and treatment of medical conditions typical of older people [17], such as OA for which the literature is still limited.

### Mediterranean diet and osteoarthritis: the epidemiological evidence

Increasing epidemiological literature is showing the importance of a higher MedDiet adherence on OA outcomes (Table 1). In the context of the Osteoarthritis Initiative, in our previous large epidemiological study including almost 5,000 participants with knee OA or at higher risk of developing this condition, participants with a higher adherence to MedDiet had a significantly lower prevalence of knee OA compared to those with lower adherence [18]. This association was still present after adjusting for several potential confounders, indicating a likely beneficial effect of MedDiet for knee OA [18]. Similarly,^1^ in the same study, analyzing magnetic resonance parameters, higher adherence to MedDiet was associated with a significant improvement in knee cartilage indexes, even after adjusting for potential confounding factors [19]. Finally, in the same context, better knee OA outcomes were also reported, considering a prospective association. Briefly, during a mean follow-up period of 4 years, participants who were more highly adherent to a MedDiet (fifth quintile) reported lower risk of pain worsening (relative risk = 0.96; 95% CI: 0.91–0.999) compared to those with a lower adherence [20]. Moreover, higher adherence to MedDiet was associated with a 9% lower risk for symptomatic forms during follow-up [20]. Altogether, these epidemiological findings suggest a beneficial role of MedDiet on OA, particularly when affecting the knee.

**Table 1:** Summary characteristics of included studies and results

Study	Participant, Demographics	Countries	Study design	Outcomes	Results
Veronese et al., 2017	4538 participants 45–79 year age range 57,6% with OA 58% females-42% males	Italy	Retrospective study	To investigate whether adherence to a Mediterranean diet is associated with lower prevalence of knee OA	Participants with a higher adherence to Mediterranean diet had a significantly lower prevalence of knee OA compared to those with lower adherence (Q4: 25.2% vs. Q1: 33.8%; *p* < 0.0001)
Veronese et al., 2018	7183 participants 45–79 year age range participants with OA not reported 60% females-40% males	Italy	Retrospective study	To investigate the adherence to a MedDiet and morphological parameters of knee joint cartilage	Mean cartilage thickness (*p* = 0.049) Volume of cartilage (*p* = 0.02)
Veronese et al., 2019	4330 participants 45–79 year age range 82% with OA 58% females-42% males	Italy	Retrospective study	To investigate whether higher adherence to Mediterranean diet is associated with lower risk of radiographic OA	participants who were more highly adherent to a Mediterranean diet (Q5) reported lower risk of pain worsening (relative risk, RR = 0.96; 95%CI: 0.91–0.999) compared to those in Q1
Dyer et al., 2017	99 participants 31–90 year age range 100% with OA 83% females-17% males	United Kingdom	Prospective study	To investigate the effects of MediDiet on perceptual, functional and serum biomarkers in subjects with OA.	No differences between groups in the response of any Arthritis Impact Measurement Scale components and most biomarkers (*p* > 0.05), except the pro-inflammatory cytokine IL-1α, which decreased in the DIET group (~ 47%, *p* = 0.010).
Davison et al., 2015	45 participants age range not reported 100% with OA sex differences not reported	United Kingdom	Prospective study	To assess whether following a Mediterranean type diet would moderate the physiological and perceptual symptoms of OA	Participants in the exposure group demonstrated lower levels of serum cartilage oligomeric matrix protein from pre- to post-intervention (pre- = 14·1 ± 4·9 U/L; post- =13·1 ± 5·2 U/L, *p* > 0·05)

## Intervention studies of mediterranean diet in osteoarthritis

Very few intervention studies explored the role of MedDiet on knee OA (Table 1). Probably, the most known is the randomized controlled trial of Dyer et al. [21]. In this study, which included 99 patients with various forms of OA, participants assigned to the MedDiet over a 16-week follow-up period experienced modest improvements in quality of life, as measured by the Short Form-12. Additionally, related outcomes showed slight enhancements, including scores on the Western Ontario McMaster University Index (WOMAC) and the Arthritis Impact Measurement Scale (AIMs). The 16 weeks dietary intervention was associated with improvement on knee and hip mobility, modest reductsion in cartilage degradation, reductions in inflammatory biomarkers as well as overall pain reductions. A pilot study, published only as a conference abstract, reported that four months of MedDiet in OA patients resulted in a significant improvement of OA biomarkers and a modest reduction in cartilage degradation. Finally and more recently, Sadeghi et al. among 129 Iranian patients with knee OA, showed that pain significantly decreased in the MedDiet group compared with the low-fat and regular diet groups and that physical function was significantly improved in the MedDiet group.

Despite the inherent methodological limitations, these small intervention studies suggest that MedDiet could be a feasible and safe intervention for people affected by this condition that could be associated with pharmacological interventions typical of OA.

## The biomolecular processes of mediterranean diet in protection against osteoarthritis

Several physiological descriptions could explain why several components of MedDiet might protect against OA. Even if OA is usually considered a degenerative joint disease, it is not simply a process of consumption, because it involves inflammation, biochemical changes, and abnormal joint remodeling. Inflammatory molecules and enzymes break down cartilage, while bone spurs and synovial fluid alterations further contribute to joint damage. Additionally, the body’s attempts to repair cartilage often result in poor-quality tissue, highlighting OA’s complexity beyond simple tissue consumption [22] Chronic low-grade inflammation, which has been defined as a fundamental mediator of OA pathogenesis and inflammation of joints in OA, is different from that in rheumatoid arthritis: it is chronic, low grade and mediated by innate immunity [23]. It was demonstrated that higher serum levels of the C-reactive protein, a marker of inflammation, are prognostic of OA advance [24]. Several studies have revealed how synovial inflammation, or synovitis, in OA, are strictly connected with improved severity of joint symptoms, cartilage loss, reduction of mobility, and higher radiographic grades of disease [25, 26], and even systemic inflammation is connected to OA advance [27]. On the other side, it is well known that metabolic syndrome, characterized by obesity, hyperglycemia, hypertension, insulin resistance, and dyslipidemia, is linked with a low and high-grade systemic inflammation [28], triggered by the excretion of proinflammatory adipokines and cytokines, which both cause joint deterioration during OA [29]. OA inflammation manifests as a multifaceted pathophysiological process involving various cell and tissue types both within and outside the joint [30]. Limited inflammation, confined in space and time, serves a beneficial role in tissue healing and reinstating balance; however, unchecked, poorly regulated inflammation, persisting without resolution, becomes detrimental and forms the foundation of chronic inflammatory conditions [31]. Various biomolecular processes could explain why several components of MedDiet might protect against OA. Even if OA is usually considered a degenerative joint disease, it is not simply a process of bone destruction [22]. Chronic low-grade inflammation, which has been defined as a fundamental mediator of OA pathogenesis and inflammation of joints in OA, appears different from that in rheumatoid arthritis: it is mediated by innate immunity [23]. It has been demonstrated that higher serum levels of the C-reactive protein, a marker of inflammation, are prognostic of OA advance [24]. Many studies have revealed how synovial inflammation, or synovitis, in OA, are strictly connected with increased severity of joint symptoms, cartilage loss, reduction of mobility, and higher radiographic grades of disease [25, 26], and even systemic inflammation is connected to OA advance [27]. On the other side, it is well known that Metabolic Syndrome (MS), characterized by obesity, hyperglycemia, hypertension, insulin resistance, and dyslipidemia, is linked with a low grade systemic inflammation [28], triggered by the excretion of proinflammatory adipokines and cytokines, which both cause joint deterioration during OA [29]. There are also other acute conditions that could cause a state of inflammation strictly connected with OA, such as car accidents, falls and sport injuries [32], or other forms of arthritis (specifically inflammatory arthritis), such as rheumatoid arthritis, gout and psoriatic arthritis [33]. Several mechanisms of MedDiet are suggested to be protective in OA. In this review we will discuss the most important, i.e., antioxidant activity and anti-inflammatory capacities. We analyze the bioactive components of MedDiet, with potential to control the metabolic abnormalities perpetuated in the context of MS (dietary strategies against MS) and the favorable outcomes that the maintenance of body weight entails (preservation of body weight).^2^ We identified these four topics, as having the greatest impact, because they appear to have an important role in the prevention of aging and age-related diseases such as OA [34]. It is well known how OA, even if affects individuals of all ages, represents a real burden for older people: an estimated 240 million individuals worldwide have symptomatic OA, including 10% of men and 18% of women age 60 and older [35]. A continuous state of Low-grade inflammation, commonly defined as inflammaging, describes the senescence of immune system in older people [36]. The four topics previous reported are main causes of inflammaging [37] and halting the senescence of the immune system could serve as a promising strategy to mitigate the adverse processes implicated in osteoarthritis [38].

### Antioxidant activity

Regular consumption of MedDiet is connected to higher levels of vitamin C detected in plasma and to decreased levels of F2-isoprostane, indicator of oxidative stress in healthy individuals [39]. MedDiet constituents, such as hydroxytyrosol, resveratrol and oleuropein, have antioxidant actions, constraining the initiation of transcription factors like nuclear factor kappa-light-chain-enhancer of activated B cells (NF-kB), activating protein-1 and reducing the manifestation of endothelial adhesion molecules [40]. In addition, polyphenols of olives offer safety against oxidative stress by decreasing the production of reactive oxygen species [41].

### Dietary strategies against MS

The bioactive constituents of the MedDiet hold promise in managing the metabolic irregularities sustained in the context of MS. This syndrome is a cluster of metabolic dysregulation including abdominal obesity, dyslipidemia, elevated blood pressure levels and impaired glucose tolerance [42]. MS represents a remarkable risk factor for mortality from cardiovascular disease [43] and several studies showed association of MS factors with OA, such as high glucose levels, hypertension, and hypercholesterolemia [44, 45]. The negative role of MS in OA, could easily be explained with the cumulative and negative impact of metabolic diseases. Obesity impacts joint tissues through increased loading, leading to chronic mechanical stress on lower limb joints. Additionally, it disrupts joint equilibrium due to systemic inflammation (meta-inflammation), involving adipokines, cytokines, and free fatty acids [44]. Type 2 diabetes exposes joint tissues to chronic high glucose levels, leading to heightened oxidative stress, cytokine release, proteolytic enzyme activity, and accumulation of advanced glycation end products in joint tissues. Synovial tissue from diabetic patients exhibits an insulin-resistant phenotype, potentially exacerbating the catabolic effects of local inflammation [46]. Dyslipidemia, characterized by high oxidized-LDL, promotes ectopic bone formation and synovial inflammation via transforming growth factor-beta activation and macrophage stimulation [47]. Finally, hypertension may induce vascular ischemia in subchondral tissues: venous obstruction of vessels in the subchondral bone, contracted venous circulation in cartilage, hypercoagulability and hypertension could lead to subchondral bone ischemia, decreased source of nutrients and oxygen to the cartilage, causing the death of cells [48].

### Preservation of body weight

Obesity represents one of the most important risk factors and it is also a predictor for the progression of OA [49]; for example, the Framingham Study demonstrated how obesity leads to knee OA. Overweight and obesity increase the risk for OA via chronic, low-grade inflammation as well as by biomechanical joint overloading. MedDiet represents a great source of soluble and insoluble dietary fibers, which higher consumption can improve satiety and decrease hunger in comparison with a low fiber intake, resulting in good body weight regulation [50].

It is important to underline the action of some bioactive components on the pathways of appetite regulation i.e. hormonal regulation, gut-brain axis. A key role is played by ghrelin and leptin. Ghrelin originates from the gastrointestinal tract and acts on specific areas of the hypothalamus to stimulate hunger sensations [51]. Both sympathetic and parasympathetic pathways contribute significantly to signaling our brain about hunger cues [52]. Consequently, ghrelin interacts with the growth hormone secretagogue receptor-1a to enhance feelings of hunger and anticipation of food [53]. On the other side, leptin is perhaps best understood as the opposite of ghrelin, acting as the body’s satiety signal [52]. The expression of the leptin receptor b, is more pronounced in the central nervous system (CNS), with research indicating that leptin’s action on the CNS alone is adequate to decrease blood glucose levels. Leptin receptors predominantly exert a GABAergic influence in various nuclei within the hypothalamus, such as the ventromedial nucleus, dorsomedial nucleus, lateral hypothalamus, and arcuate nucleus [54].

Ghrelin has an important role in the inhibition pro-inflammatory cytokines from monocytes, T-cells, and macrophages. It also inhibits leptin-induced pro-inflammatory cytokine expression including interleukin (IL)-1β, IL-6, and tumor necrosis factor (TNF)-α in human T lymphocytes and monocytes [55].

Inflammatory actors across the gut-brain axis have crucial roles: neurons and glial cells within the enteric nervous system actively engage in intestinal immunity. Alongside their role in supporting enteric neurons, enteric glia provide protection to the intestinal barrier by releasing ligands for the REarranged during Transfection receptor, which in turn stimulate the production of IL-22 by ILC3 cells [56]. Tissue-protective macrophages in the muscularis mucosa closely interact with enteric neurons via bone morphogenetic protein (BMP)-2-BMP receptor signaling, fostering neuronal viability and constraining inflammation-induced bowel dysmotility [57]. It has been demonstrated how the Western diet, typified by high intake of saturated fat and sugar, disrupts the expression of intestinal barrier markers, diminishes glucagon-like peptide 1 derived from enteroendocrine cells, provokes inflammation in the mediobasal hypothalamus via microglial release of IL-1β, IL-6, and TNF-α, instigates resistance to the appetite-regulating hormone leptin centrally, and fosters anxiety-like behavior [58, 59]. Instead, MeDdiet and its components revealed a crucial role against inflammation in gut-brain axis, for example short chain fatty acids (SCFAs) are immunomodulatory, stimulating G-protein-coupled receptor, promoting innate immune responses, and inducing regulatory T cells [60]. Supplementation with SCFAs could protected against high-fructose diet-associated neuroinflammation and neuronal loss by alleviating intestinal barrier impairment [61]. A German meta-analysis showed how MedDiet pattern was significantly associated with a reduction of IL-6 and IL-1β and even the biomarkers CRP, IL-8, and TNF-α also showed a tendency to decrease as a consequence of this diet [62].

### Anti-inflammatory capacities

It was showed that inflammatory alterations are detected in synovial lining and typical radiographic aspects are the injury of cartilage, development of osteophytes and alterations in subchondral bone [63]. Mediators of these alterations are: macrophages, the proinflammatory cytokines, like TNF-alpha, nitric oxide, metalloproteinases, IL-1a, IL-1b, IL-15, IL-17, IL-8 and prostaglandin E2 [64]. In the traditional MedDiet there is a high consumption of extravirgin olive oil, fruits, vegetables, legumes, whole grains and fish. Those are sources of monounsaturated fatty acids, n-3 polyinsaturated fatty acid (PUFAs), ascorbic acid, carotenoids, vitamin E, and phytochemicals with anti-inflammatory properties, that are crucial for protection against inflammatory and oxidative processes [64]. Several studies showed the considerable anti-inflammatory capacities of non-fat and fat constituents of MedDiet, through the manifestation of proinflammatory genes, control of arachidonic acid (AA) sequence and the role of immune system [65]. AA is produced in human body from the linoleic acid (n-6 PUFA). Production of AA can decrease inflammation through regulation of eicosanoid synthesis, that modulates AA in the tissues [65]. On the other side, non-fatty elements of virgin olive oil have a significant effect against proinflammatory cytokines [66]; other compoents content arginine, dietary fibers, magnesium, phenolic compounds and other phytochemicals, which have shown crucial anti-inflammatory properties [67].

## Future directions

In this review, we have summarized the current literature about the importance of following MedDiet in order to help preventing and treating OA. As observed by Zeng et al., in their systematic review about the effects of dietary patterns and food groups on symptomatic OA, the body of evidence supporting MedDiet is still limited, suggesting that further research is needed to corroborate the estimated effect at a high certainty of evidence. This point indicates the necessity of further studies, particularly of intervention, to promote MedDiet in order to help preventing OA. Moreover, we believe that long-term prospective studies are warranted to assess the sustained benefits of adopting a MedDiet pattern on OA outcomes, including the risk of joint replacement surgery and other OA-related complications.

Among the future directions of research, an important aspect is to elucidate the underlying mechanisms by which components of the MedDiet exert protective effects against OA development and progression. Molecular and cellular studies can provide insights into the anti-inflammatory, antioxidant, and chondroprotective properties of MedDiet components, such as polyphenols, omega-3 fatty acids, and other bioactive compounds. Similarly, advanced imaging techniques, biomarkers analysis, and metabolomics studies may help identify biomarkers of dietary intake and metabolic pathways associated with OA risk and progression, providing more precise mechanistic links between diet, inflammation, and joint health.

Finally, future research should explore the concept of personalized nutrition in OA management, considering individual variations in dietary preferences, genetics, gut microbiota, and metabolic profiles. Nutrigenomics and precision medicine approaches can help tailor dietary recommendations to individual needs and optimize therapeutic outcomes.

## Conclusions

MedDiet holds promise as a dietary approach for preventing and managing OA, but further research is needed to elucidate its mechanisms of action, optimize dietary interventions, and translate research findings into clinical practice. Collaborative efforts among researchers, healthcare providers, policymakers, and patients are essential to advance our understanding of the role of diet in OA and develop evidence-based strategies to promote joint health and improve outcomes for individuals living with OA.

There is a trend of decline of adherence to the MD in many Mediterranean countries including Italy due to socioeconomic influence A future goal should be to guide the public on how the Mediterranean diet’s health benefits, such as reduced risk of chronic diseases, can outweigh economic concerns. By promoting local, seasonal produce and focusing on affordable staples like legumes and grains, it should be illustrated practical, cost-effective ways to adopt this diet. Emphasizing long-term healthcare savings and simple meal planning can make the Mediterranean diet both accessible and sustainable.

## Data Availability

No datasets were generated or analysed during the current study.

## Footnotes

1 prof qui dovremmo capire se togliere questo in the same study, perchè si parla di un altro studio in realtà rispetto al precedente.

2 Non so se sta parte in cui parliamo di questi 4 topics va scritta meglio, perchè sono due prima e due qui più sotto.
